# Effect of Gamma-Oryzanol as Therapeutic Agent to Prevent Cardiorenal Metabolic Syndrome in Animals Submitted to High Sugar-Fat Diet

**DOI:** 10.3390/nu9121299

**Published:** 2017-11-29

**Authors:** Fabiane Valentini Francisqueti, Igor Otávio Minatel, Artur Junio Togneri Ferron, Silméia Garcia Zanati Bazan, Vanessa dos Santos Silva, Jéssica Leite Garcia, Dijon Henrique Salomé de Campos, Ana Lúcia Ferreira, Fernando Moreto, Antonio Carlos Cicogna, Camila Renata Corrêa

**Affiliations:** 1 São Paulo State University (Unesp), Medical School, Botucatu 18618-687, Brazil; fabiane_vf@yahoo.com.br (F.V.F.); artur.ferron@gmail.com (A.J.T.F.); sgzanati@fmb.unesp.br (S.G.Z.B.); vangynera@gmail.com (V.d.S.S.); jessleitegarcia@gmail.com (J.L.G.); dijoncampos@gmail.com (D.H.S.d.C.); ferreira@fmb.unesp.br (A.L.F.); fer_moreto@yahoo.com.br (F.M.); cicogna@fmb.unesp.br (A.C.C.); 2 São Paulo State University (Unesp), Institute of Biosciences, Botucatu 18618-689, Brazil; igorminatel@hotmail.com

**Keywords:** high sugar-fat diet, obesity, cardiac dysfunction, renal disease, gamma-oryzanol, Cardiorenal Metabolic Syndrome

## Abstract

Background: The high consumption of fat and sugar contributes to the development of obesity and co-morbidities, such as diabetes, and cardiovascular and kidney diseases. Different strategies have been used to prevent these diseases associated with obesity, such as changes in eating habits and/or the addition of dietary components with anti-inflammatory and anti-oxidant properties, such as gamma-oryzanol (γOz) present mainly in bran layers and rice germ. Methods: Animals were randomly divided into four experimental groups and fed ad libitum for 20 weeks with control diet (C, *n* = 8), control diet + γOz (C + γOz, *n* = 8), high-sugar and high-fat diet (HSF, *n* = 8), and high-sugar and high-fat diet + γOz (HSF + γOz, *n* = 8). HSF groups also received water + sucrose (25%). The dose of γOz was added to diets to reach 0.5% of final concentration (*w*/*w*). Evaluation in animals included food and caloric intake, body weight, plasma glucose, insulin, triglycerides, uric acid, HOMA-IR, glomerular filtration rate, protein/creatinine ratio, systolic blood pressure, and Doppler echocardiographic. Results: Animals that consumed the HSF diet had weight gain compared to group C, increased insulin, HOMA, glucose and triglycerides, there were also atrial and ventricular structural alterations, deterioration of systolic and diastolic function, decreased glomerular filtration rate, and proteinuria. Gamma-oryzanol is significantly protective against effects on body weight, hypertriglyceridemia, renal damage, and against structural and functional alteration of the heart. Conclusion: Gamma-oryzanol shows potential as a therapeutic to prevent Cardiorenal Metabolic Syndrome.

## 1. Introduction

Obesity is considered a public-health problem, with both direct and indirect costs [1,2,3], such as diabetes, cardiovascular disease and hypertension, workdays lost, physician visits, disability pensions, and premature mortality [4]. In recent decades, there has been a change in eating behavior where the preference for palatable food has prevailed. The intake of sugar-sweetened beverages and fat, especially from industrialized foods, has clearly increased around the world [5]. However, the consumption of these dietary components has exceeded recommended daily levels, contributing to the development of obesity and its comorbidities, such as diabetes, cardiovascular and kidney diseases, and negative health consequences linked to modern dietary habits [6,7,8]. All these factors contribute to the strict relationship between obesity and the early development of a constellation of diseases named Cardiorenal Metabolic Syndrome (CRS), a terminology that explains co-existing heart and kidney disease, characterized clinically by: impaired coronary blood flow, impaired diastolic relaxation, impaired ischemic reconditioning, renal hyper-filtration, proteinuria, glomerular sclerosis, tubule-interstitial fibrosis, and decreased GFR (glomerular filtration rate) [9].

The pathophysiology of CRS can be elucidated by four connectors: the renin-angiotensin-aldosteron system (RAAS), the sympathetic nervous system (SNS), inflammation, and nitric oxide/reactive oxygen species (ROS) balance [10,11,12]. The literature reports some experimental models to study the cardio-renal syndrome. However, these models use invasive methods, such as: ligation of the left coronary artery, unilateral nephrectomy and sub-nephrectomy [13,14,15]. An alternative model, which does not require surgical intervention, is the model of adriamycin-induced renal damage [16]. Thus, it is necessary to develop a non-invasive model which mimics the habits of the population and reproduces the clinical situation observed.

Considering this situation, it is important to seek effective alternatives to treat or to prevent this disease. A wide range of medicines is currently used to treat obesity and related disorders; however, many adverse effects and high rates of secondary failures have been associated with these [17]. Thus, finding natural drugs has now become the focus of scientists and researchers. Gamma-oryzanol (γOz) is a compound present mainly in the bran and germ of rice [18,19,20], a widely available and inexpensive food source that has been investigated due to its many biological activities, including anti-oxidative and anti-inflammatory effects [21,22,23], which led us to hypothesize that the compound is able to prevent CRS. Therefore, the aim of this study was to test the therapeutic potential of gamma-oryzanol to prevent Cardiorenal Metabolic Syndrome in animals with a high sugar-fat diet.

## 2. Materials and Methods

### 2.1. Animals and Experimental Protocol

All of the experiments and procedures were approved by the Animal Ethics Committee of Botucatu Medical School (1150/2015) and were performed in accordance with the National Institute of Health’s Guide for the Care and Use of Laboratory Animals. Male Wistar rats (±187 g) were kept in an environmental controlled room (22 °C ± 3 °C; 12 h light-dark cycle and relative humidity of 60 ± 5%) and randomly divided into 4 experimental groups. Over 20 weeks the animals received: control diet (C, *n* = 8), control diet + gamma-oryzanol (C + γOz, *n* = 8), high sugar-fat diet (HSF, *n* = 8), and high sugar-fat diet + gamma-oryzanol (HSF + γOz, *n* = 8). HSF groups also received water + sucrose (25%). The diets and water were ad libitum.

### 2.2. Gamma-Oryzanol

γOz is the major bioactive compound of rice bran [18,19,20], and has been investigated due to its antioxidant and anti-inflammatory activities [21,22,23]. The compound was purchased from Tokyo Chemical Industry Co., Ltd. (Toshima, Kita-ku, Tokyo) (lot.5ZZYLPJ). To simulate its regular way of consumption and due to its nonpolar characteristics, the compound was added to diets to reach 0.5% of final concentration (*w*/*w*). Diets and the period of treatment were based on the work of Son et al. [17] and on the daily consumption of rice of an adult individual in Brazil, according to data from the Family Budget Survey (POF) 2008–2009 [24].

### 2.3. Diets

The diets used in this study were designed in our laboratory. The HSF diet contained soybean meal, sorghum, soybean peel, dextrin, sucrose, fructose, lard, vitamins, and minerals, plus 25% sucrose in drinking water. Control diet contained soybean meal, sorghum, soybean peel, dextrin, soy oil, vitamins, and minerals. The nutrients and nutritional composition of each diet are presented in Table 1.

### 2.4. Nutritional Analysis

The nutritional profile was evaluated according to the following parameters: food and caloric intake, and body weight. Food consumption was measured daily and body weight weekly. Caloric intake was determined by multiplying the energy value of each diet (g × Kcal) by the daily food consumption. For the HSF group, caloric intake also included calories from water (0.25 × 4 × mL consumed).

### 2.5. Metabolic and Hormonal Analysis

After 12-h fasting, blood was collected from the tail and the plasma was used to measure insulin and biochemical parameters. Glucose concentration was determined by using a glucometer (Accu-Chek Performa; Roche Diagnostics, Indianapolis, IN, USA); triglycerides and uric acid were measured with an automatic enzymatic analyzer system (Chemistry Analyzer BS-200, Mindray Medical International Limited, Shenzhen, China). The insulin level was measured using the enzyme-linked immunosorbent assay (ELISA) method using commercial kits (EMD Millipore Corporation, Billerica, MA, USA). The homeostatic model of insulin resistance (HOMA-IR) was used as an insulin resistance index, calculated according to the formula: HOMA-IR = (fasting glucose (mmol/L) × fasting insulin (µU/mL))/22.5 [25].

### 2.6. Renal Function

Renal function was evaluated by measurements of plasma and urine. At 24 h urine was collected from the metabolic cages to measure the excretion of creatinine and the total protein. The urea and creatinine content of the plasma were measured. All analyses were performed with an automatic enzymatic analyzer system (Chemistry Analyzer BS-200, Mindray Medical International Limited, Shenzhen, China)). The glomerular filtration rate (GFR) = ((urine creatinine × flux)/plasma creatinine) was also calculated and the protein/creatinine ratio that reflects proteinuria was considered as a marker of kidney function [26].

### 2.7. Systolic Blood Pressure

Systolic blood pressure (SBP) evaluation was assessed in conscious rats by the non-invasive tail-cuff method with a NarcoBioSystems^®^ Electro-Sphygmomanometer (International Biomedical, Austin, TX, USA). The animals were kept in a wooden box (50 × 40 cm) between 38–40 °C for 4–5 min to stimulate arterial vasodilation [27]. After this procedure, a cuff with a pneumatic pulse sensor was attached to the tail of each animal. The cuff was inflated to 200 mmHg pressure and subsequently deflated. The blood pressure values were recorded on a Gould RS 3200 polygraph (Gould Instrumental, Valley View, OH, USA). The average of three pressure readings was recorded for each animal.

### 2.8. Structural and Functional Cardiac Function by Echocardiogram

Doppler echocardiographic evaluation was performed by a single examiner at the 20th week. Animals were anesthetized with ketamine (50 mg/kg, i.p.) and xylazine hydrochloride (1 mg/kg, i.p.). After trichotomy of the anterior chest region, the animals were placed in slight left lateral decubitus for the exam. The equipment used was model Vivid S6 (General Electric Medical Systems, Tirat Carmel, Israel) with a multifrequency ultrasonic transducer 5.0 to 11.5 MHz. To implement structural measurements of the heart, the images were obtained in one-dimensional mode (M-mode) guided by the images in two-dimensional mode with the transducer in the parasternal position, minor axis. Left ventricular (LV) evaluation was performed by positioning the cursor M-mode just below the mitral valve plane at the level of the papillary muscles. The images of the aorta and left atrium were obtained by positioning the M-mode course to plan the level of the aortic valve. The following cardiac structures were used to analyze cardiac morphology: left ventricular diastolic diameter (LVDD); left ventricular systolic diameter (LVSD); left atrium (LA); LA/AO; relative wall thickness (RWT). The LV systolic function was assessed by the following parameters; endocardial fractional shortening (FS%) ((LVDD − LVSD)/LVDD) × 100; posterior wall shortening velocity (PWSV). The LV diastolic function was evaluated using the following indices: peak velocity of early diastolic filling (E wave). The study was supplemented by evaluation by tissue Doppler systolic displacement (S′), early diastolic (E′) and late (A′) of the mitral annulus (arithmetic average travel speeds of lateral and septal walls), and E/A and E/E′ wave ratios.

### 2.9. Statistical Analysis

Data are presented as means ± standard deviation (SD) or medians (interquartile range). Differences among the groups were determined by two-way analysis of variance. Statistically significant variables were subjected to the Tukey post-hoc test to compare all the groups. Statistical analyses were performed using Sigma Stat for Windows Version 3.5. (Systat Software, Inc., San Jose, CA, USA). A *p* value of 0.05 was considered as statistically significant. Power calculations for the outcome variables were above 80%.

## 3. Results

### 3.1. Nutritional, Metabolic, and Hormonal Analysis

Nutritional, metabolic, and hormonal analyses are showed in Table 2. The HSF group exhibited a higher body weight value than control group. Additionally, γOz prevented weight gain in HSF + γOz group. Glucose, insulin, and HOMA-IR were higher in HSF and HSF + γOz, when compared to control groups. The triglycerides level was higher in the HSF group and lower in the HSF + γOz group.

### 3.2. Renal Function

**Figure 1:**
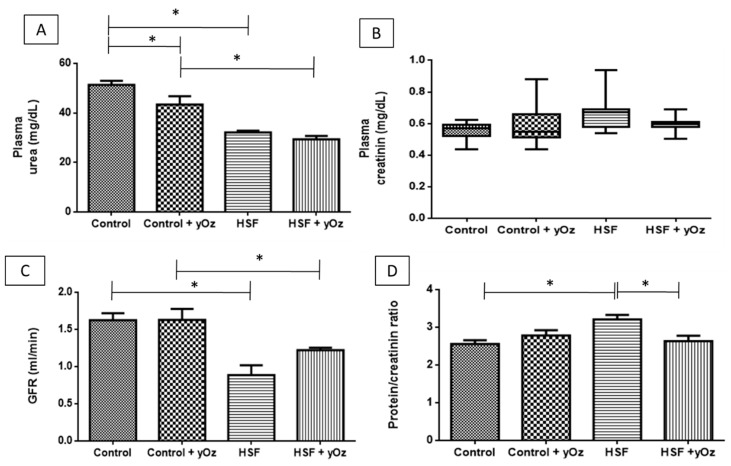
Renal function of animals. (**A**) plasma urea (mg/dL); (**B**) plasma creatinin (mg/dL); (**C**) glomerular filtration rate—GFR (mL/min); and (**D**) protein/creatinin ratio (proteinuria). Data are expressed as means ± standard deviation. Comparison by two-way ANOVA with Tukey post-hoc. HSF—high sugar fat diet. γOz—Gamma-oryzanol. * statistically different.

Plasma urea was lower in the HSF and HSF + γOz groups, with no influence of γOz (Figure 1A). No significant difference among the four groups was observed for plasma creatinine (Figure 1B). Considering the renal function parameters, we can note that HSF diet worsened the GFR in HSF groups (Figure 1C). However, in HSF + γOz this ratio was slightly less impaired. The HSF group presented higher proteinuria when compared to C group, therefore γOz was clearly efficient in the prevention of this condition (Figure 1D).

### 3.3. Cardiac Parameters and Systolic Blood Pressure

The values of cardiac function and systolic blood pressure are presented in Table 3. The diet promoted an increase in systolic blood pressure, left atrium, relative left ventricular thickness and deterioration of systolic function, visualized by the decrease in the ejection fraction, of S′ and PWSV. Regarding diastolic function, although the diet did not alter the E wave and E/A wave, it promoted E/E′ increase. Although the γOz did not change any variables in the control group, it led to a decrease in LA, and in the relative thickness of the wall, and prevented the deterioration of systolic and diastolic function.

## 4. Discussion

All analyzes performed in this experiment were made with animals in vivo, because it was an intermediate evaluation in the experimental protocol. The aim of this study was to present therapeutic effects of γOz on diseases related to the consumption of a high sugar-fat diet, with special focus on cardiac remodeling and renal disease. According to the literature, the simple carbohydrate is part of modern eating habits and is one of the main causes of the development of obesity, comorbidities, cardiac dysfunction, and renal disease [28,29,30] due to its lower molecular structure, high glycemic index, and fast absorption, leading to a higher fat deposition [31,32]. Saturated fatty acid is also largely consumed by the population and its role in inducing fat accumulation, metabolic disorders, and renal and cardiac dysfunction is already described [30]. Corroborating these data, HSF diet promoted metabolic, hormonal, cardiovascular, and renal changes, and increased body weight. The set of complications and comorbidities presented in this study are relevant because they are usually found in studies involving experimental models genetically modified for obesity [33,34,35]. Therefore, we can infer that the diet created for this study was able to mimic modern eating habits, as well as the development of associated diseases.

Although this paper presents preliminary results, it is possible to note the efficiency of γOz to prevent some disorders related to high sugar-fat diet consumption. The levels of γOz in brown rice range on average from 10–150 mg/100 g and the consumption of γOz by rats in this study was approximately 127 mg/day in Control + γOz, and 50 mg/day in HSF + γOz. Taking into account that in Brazil and others countries, where rice is a popular foodstuff and that people consume at least 100 g/day of this grain, the health benefits observed in this study can plausibly be attained in humans.

Literature reports that γOz increases the elimination of fecal fat by inhibiting intestinal lipase, which impairs the degradation of ingested triglycerides to fatty acid and consequently reduces intestinal absorption, levels of serum triglycerides, and also storage in adipose tissue [22,36,37]. Our findings support this concept since the triglycerides levels were lowest in the HSF + γOz group, and inhibited weight gain, since the body weight in HSF + γOz was similar to the control group. However, some deeper pathways may also be responsible for this outcome. Immature adipocytes require lipid uptake and γOz reduce the activities of glycerol-3-phosphate dehydrogenase (GPDH), fatty acid synthase (FAS), fatty acid binding protein 4 (Fabp4), and sterol regulatory element-binding protein-1c (SREBP-1c), important lipogenic enzymes. In addition, the γOz is also able to inhibit PPARγ (proliferator-activated receptor γ) and C/EBPs (CCAAT-enhancer-binding proteins)—key factors involved in adipocytes differentiation and adipogenesis [38].

In relation to glucose metabolism parameters, the compound was not effective in preventing the changes, because the values of glucose, insulin and HOMA-IR index were higher in both groups that received HSF diet. Due the scarcity of literature addressing γOz and prevention of comorbidities in obese rats, we found only two studies by Wang et al. and Justo et al. [23,37], which showed reduced glycemia, insulin, and HOMA-IR with γOz treatment. The reason for the difference between this response and our data may be that these studies used genetically obese animals, and also a lower concentration of simple carbohydrates in their diet compared to ours. Regarding systolic blood pressure, γOz did not show efficacy in reducing values in either HSF group. Contrary to our results other researchers reported a reduction in blood pressure, but in these studies obese animals were supplemented with rice bran, which contains γOz, and also ferulic acid, the main component responsible for the reduction in this parameter [37,39].

According to Sowers [9] there is a cluster of interactive cardiac and renal risk factors, including overweight/obesity, hypertension, insulin resistance/hyperinsulinemia and dyslipidemia, microalbuminuria and/or reduced renal function, all of which constitute Cardiorenal Metabolic Syndrome (CRS). All these changes were present in HSF group. The protection afforded by γOz in both organs occurred even with the maintenance of high blood pressure, blood glucose, and insulin levels, as showed by the maintenance in proteinuria and cardiac function in the HSF + γOz group. However, the cardiac and renal function impairment in the untreated HSF group may be due to the intrinsic mechanisms of obesity, and the probability of lipotoxicity, which is characterized by the increased flow of lipids to non-adipose organs when the adipose tissue capacity store is exceeded, resulting in ectopic fat deposits [40,41]. The inference that lipotoxicity was the main cause of comorbidities was due to the fact that the animals treated with γOz did not show hypertriglyceridemia or obesity, but maintained alterations in three risk factors: high glucose, insulin, and blood pressure. In this condition, fatty acids are more oxidized, stimulating an increased release of reactive species. Moreover, when this oxidation capacity is exceeded, there is an increase in non-oxidative intracellular pathways, generating cytotoxic derivatives, such as ceramide and diacylglycerol. These situations are responsible for the development of inflammation [42] and oxidative stress [43,44], also related to organ dysfunction. Our most important finding was to evidence the effect of the isolated compound which prevents the development of CRS. Therefore, as previously described [22,45,46], we can suggest that γOz acts as an anti-inflammatory and an anti-oxidant, preventing lipid peroxidation and avoiding lipotoxicity, which is associated with mitochondrial dysfunction and the formation of cellular ROS. In addition, the compound may be useful in preventing the development of the inflammatory process, reducing the amount of pro-inflammatory cytokines, such as TNF-α (tumor necrosis factor α), IL-6 (interleukin 6), and IL-1β [38,47].

## 5. Conclusions

In summary, γOz prevented weight gain, hypertriglyceridemia, systolic and diastolic dysfunction, and renal damage. These findings provide important information about the use of bioactive compounds as alternative therapeutics for obesity, related disorders, and CRS; however, more studies to investigate the causal mechanisms are necessary, although the effect of γOz in preventing the clinical condition of Cardiorenal Metabolic Syndrome was remarkable.

## Figures and Tables

**Table 1:** Diet composition and nutritional values.

Components	Control	HSF
Soybean meal (g/kg)	335	340
Sorghum (g/kg)	278	80
Soy hulls (g/kg)	188	116
Dextrin (g/kg)	146	20
Sucrose (g/kg)	-	80
Fructose (g/kg)	-	180
Soybean oil (g/kg)	14	-
Lard (g/kg)	-	154
Minerals (g/kg)	25	25
Salt (g/kg)	4	8
Nutritional values		
Protein (% of ingredients)	20.0	18.0
Carbohydrate (% of ingredients)	60.0	53.5
Fat (% of ingredients)	4.00	16.5
% of unsaturated	69.0	47.0
% of saturated	31.0	53.0
% Energy from protein	22.9	16.6
% Energy from carbohydrate	66.8	49.2
% Energy from fat	10.4	34.2
Energy (kcal/g)	3.59	4.35

HSF: high sugar-fat diet.

**Table 2:** Nutritional, metabolic, and hormonal analysis.

	Groups				Effects		
	Control	Control + yOz	HSF	HSF + yOz	Diet	yOz	Interaction
Final body weight	461 ± 54	475 ± 57	540 ± 48 *	447 ± 66 ^†^	0.212	0.061	0.013
Chow fed (g/day)	24.2 ± 2.4	25.4 ± 2.4	12.2 ± 2.4 *	10.1 ± 1.5 ^#^	<0.001	0.030	0.018
Water intake (mL/day)	35.0 ± 5.1	35.2 ± 6.3	43.8 ± 3.8 *	43.3 ± 6.5 ^#^	<0.001	0.280	0.236
Caloric intake (kcal/day)	86.9 ± 8.7	91.0 ± 8.6	96.8 ± 8.7	87.4 ± 9.6	0.342	0.846	0.147
Glucose (mg/dL) ^1^	85.2 (11.7)	89.7 (5.2)	115.0 (15.5) *	132.1 ± 30.9 ^#^	<0.001	0.294	0.522
Triglycerides (mg/dL)	79.9 ± 14.3	63.2 ± 14.3	113.0 ± 24.1 *	89.7 ± 24.1 ^†,#^	<0.001	0.008	0.638
Insulin (ng/mL)	2.66 ± 1.27	3.42 ± 1.62	5.85 ± 1.25 *	5.32 ± 1.93 ^#^	<0.001	0.84	0.249
HOMA-IR	23.0 ± 11.6	27.3 ± 15.2	68.7 ± 11.6 *	69.1 ± 26.7 ^#^	<0.001	0.744	0.786
Uric acid	1.08 ± 0.11	1.14 ± 0.41	1.23 ± 0.18	1.32 ± 0.19	0.198	0.311	0.619

Data expressed as means ± standard deviation. ^1^—median (interquartile range). Comparison by two-way ANOVA with Tukey post-hoc. *p* < 0.05. HSF—high sugar fat diet. γOz—Gama-oryzanol. * vs. Control; ^†^ vs. HSF; ^#^ vs. Control + γOz.

**Table 3:** Cardiac function and systolic blood pressure.

	Groups				Effect		
	Control	Control + yOz	HSF	HSF + yOz	Diet	yOz	Interaction
HR	239 ± 44	230 ± 40	280 ± 61	271 ± 50	0.008	0.737	0.884
SBP	129 ± 4	129 ± 5	137 ± 7 *	139 ± 5 ^#^	<0.001	0.915	0.815
LVDD (mm)	7.05 ± 0.30	6.74 ± 0.30	6.42 ± 0.35	7.05 ± 0.35	0.777	0.789	0.0037
LA (mm)	4.69 ± 0.23	4.63 ± 0.52	5.67 ± 0.21 *	4.76 ± 0.19 ^†^	<0.001	<0.001	<0.001
LA/AO	1.24 ± 0.09	1.18 ± 0.20	1.49 ± 0.11 *	1.24 ± 0.08 ^†^	0.696	0.950	0.024
RWT	0.44 ± 0.02	0.44 ± 0.02	0.59 ± 0.09 *	0.43 ± 0.01 ^†^	<0.001	<0.001	<0.001
FS (%)	59.8 ± 4.0	58.9 ± 4.2	53.8 ± 5.6 *	56.6 ± 4.0 ^#^	<0.001	0.197	0.439
S′m (cm/s)	5.61 ± 0.28	5.67 ± 0.27	4.92 ± 0.55 *	5.66 ± 0.33 ^†^	<0.001	<0.001	0.003
PWSV (cm/s)	60.8 ± 4.3	60.6 ± 2.4	52.8 ± 5.9 *	61.9 ± 3.6 ^†^	0.005	<0.001	0.003
E wave (cm/s)	74.3 ± 3.8	73.8 ± 4.9	76.7 ± 7.6	76.8 ± 6.1	0.102	0.748	0.484
E/A	1.72 ± 0.24	1.95 ± 0.30	1.84 ± 0.57	1.77 ± 0.19	0.339	0.262	0.239
E/E′	13.8 ± 0.8	13.8 ± 1.5	17.6 ± 3.5 *	14.2 ± 1.2 ^†^	0.002	0.008	0.041

HR—heart rate; SPB—systolic blood pressure; LVDD—left ventricular diastolic diameter; LA—left atrium; LA/AO—left atrium/aortic diameter; RWT—relative wall thickness; FS—endocardial fractional shortening; PWSV—posterior wall shortening velocity. Data expressed in mean ± standard deviation. Comparison by two-way ANOVA with Tukey post-hoc. *p* < 0.05. HSF—high sugar fat diet. γOz—Gama-oryzanol. * vs. Control; ^†^ vs. HSF; ^#^ vs. Control + γOz.
